# Management strategy and outcomes of sacrococcygeal teratoma — an Egyptian multicenter experience

**DOI:** 10.1186/s12957-023-03180-w

**Published:** 2023-09-18

**Authors:** Ahmed Elgendy, Amr Abdelhamid AbouZeid, Mohamed El-Debeiky, Mahmoud Mostafa, Mohammed Hamada Takrouney, Mohamed Abouheba, Ahmed Khairi, Sameh Shehata, Sherif M. Shehata

**Affiliations:** 1 Surgical Oncology Unit, Department of Surgery, Faculty of Medicine, Tanta University, Tanta, 31515 Egypt; 2 Pediatric Surgery Department, Faculty of Medicine, Ain Shams University, Cairo, Egypt; 3 Pediatric Surgery Unit, Department of Surgery, Faculty of Medicine, Assiut University, Assiut, Egypt; 4 Pediatric Surgery Unit, Department of Surgery, Faculty of Medicine, Alexandria University, Alexandria, Egypt; 5 Pediatric Surgery Unit, Department of Surgery, Faculty of Medicine, Tanta University, Tanta, Egypt

**Keywords:** Sacrococcygeal teratoma, Surgery, Relapse, Outcomes

## Abstract

**Objectives:**

Nationwide criteria regarding patients with sacrococcygeal teratoma (SCT) are still lacking in Egypt. We aimed to present a multicenter study regarding the management and outcomes of this tumor to evaluate our national treatment strategy.

**Methods:**

A retrospective analysis including all patients with SCT who were managed at four major Egyptian centers between 2013 and 2023. Clinical data, surgical approaches, and short- and long-term outcomes were discussed.

**Results:**

The study included 95 patients (74 were females). Antenatal diagnosis was reported in 25% of patients. Seventy-one patients (74.7%) were classified as Altman type I/II. Surgery was performed via a perineal approach in 75 patients, whereas the remaining 20 underwent a combined abdominoperineal approach. Vertical elliptical incision with midline closure was conducted in 51.5% of patients, followed by classic or modified chevron incisions. Benign mature teratoma was detected in 82% of patients. At a median follow-up of 57 months, eight patients (8.5%) had relapsed. The 5-year overall survival (OS) and event-free survival (EFS) of all patients were 94% and 91%, respectively. In the after-care monitoring, 19 patients (20%) had urinary or bowel dysfunctions. Nine of them were managed using medications. Clean intermittent catheterization was practiced in another five patients. The remaining five underwent further surgical interventions.

**Conclusion:**

Favorable outcomes were achieved in our country during the last decade. Diverse perineal incisions were performed for resection, and vertical elliptical with midline closure was the commonest. During follow-up, 20% of patients developed urological or bowel dysfunctions that required medical and surgical treatment modalities to improve their quality of life.

## Introduction

Sacrococcygeal teratoma (SCT) is a germ cell neoplasm that originates from the coccyx and contains all three germ layers [1]. Although SCT is the most common tumor in neonates, it is a rare entity with an estimated incidence of one patient in 40,000 live births with a prevalence in females [2, 3]. About 18% of all cases are presented with additional congenital anomalies [4]. In the majority of patients, the diagnosis is obvious due to the presence of a noticeable mass in the sacrococcygeal region during the neonatal period. However, patients with huge pelvic components have been referred later due to symptoms of urine retention, or constipation, or palpable pelviabdominal mass [5]. Doppler ultrasound and magnetic resonance imaging (MRI) are the current radiological modalities of choice [6].

The majority of patients have benign pathology; complete tumor resection (via perineal approach) is the cornerstone for the treatment and is associated with long-term disease-free [7]. A combined perineal and abdominal approach is crucial for lesions with intrapelvic extension. The abdominal component can be performed laparoscopically in selected patients [8]. Malignant tumors are rarer, and the administration of platinum-based chemotherapy is imperative besides surgery. Interdisciplinary management can achieve an overall survival of up to 80% [1, 9, 10]. Operative complications include hemorrhage due to injury of median sacral artery [11] and tumor rupture with subsequent recurrence [12]. Additionally, functional problems related to urological and bowel control [13] and unsatisfactory scars may be encountered during long-term follow-up [14].

Due to the paucity of SCT, single institutional series with relatively limited numbers of cases were conducted in Egypt to report the outcomes [15–17]. However, nationwide criteria regarding patients with this tumor are still lacking. Thus, the authors of this study aimed to present the first Egyptian multicenter experience regarding clinical data, management, and outcomes to assess our national treatment strategy.

## Material and methods

We retrospectively reviewed the medical records of all patients with SCT who were treated within four major Egyptian institutions (Tanta, Ain Shams, Assiut, and Alexandria) including their affiliated regional hospitals from January 2013 to January 2023. Neonatal and late-presenting cases with histopathological diagnosis of SCT were included in the analysis. Late presentation was considered as any patient presenting with SCT after 1 month of birth. The patients who were lost to follow-up were excluded from the study. The research ethics committee board of Tanta University, as the lead coordinating center, has reviewed and approved the study proposal (approval code: 36264PR189). Due to the retrospective nature of the study, the written consents of the patients’ parents were waived.

A uniform data set was designed by the first author, and then it was reviewed by the senior authors of all participating centers. The patients’ data have been retrieved from the written medical files by the authors of the four participating institutions. The retrieved data included patients’ demographics, Altman’s classification [18], imaging characteristics, surgical incisions and approaches, complications happened, pathological types, tumor recurrence, short and long-term outcomes, and survival rates. The radiological images of all included patients who were treated at the participating centers were captured by cameras and electronically stored. Wound infection, dehiscence, necrosis, or unsatisfactory cosmetic scars were reported. Broad-spectrum antibiotics (third-generation cephalosporin and metronidazole) were administered to all patients in the perioperative period, and wound inspection and careful dressing were performed daily to minimize wound infection. Additionally, postoperative bowel and urinary functions were documented. Recurrence was defined as a tumor (proved to be SCT) recurring during follow-up after its successful primary radical resection. The monitoring schedule was conducted according to institutional internal guidelines, and follow-up data were collected until May 2023. Clinical assessment, serum alpha-fetoprotein (AFP), and imaging scans were routinely performed in the follow-up visits. The overall survival (OS) and event-free survival (EFS) rates were estimated using the Kaplan–Meier method and reported at 5 years.

## Results

### Clinical characteristics

A total of 95 patients with SCT (78% females) were identified and enrolled in the analysis. The age at first presentation of all patients ranged from day 1 to 26 months. The antenatal diagnosis was reported in 25% of all patients (*n* = 24) using antenatal ultrasonography at a median gestational age of 26 weeks (range: 23–31 weeks). Intrauterine fetal intervention was not performed in any patient with antenatal diagnosis, and abortion was not permitted as per the Egyptian laws for SCT upon antenatal diagnosis. Cesarean section was the delivery mode for all patients who were diagnosed antenatally. Fifty-two patients (55%) were diagnosed at birth or during the neonatal period with a median age of 2 days (range: 1–28 days). Late presentation, after the neonatal period, was observed in 19 patients (20%). Out of them, 14 patients were diagnosed within the first year of age. The remaining 5 patients were presented after 1 year with a median age of 17.5 months. Associated congenital anomalies were detected in 15 patients (15.8%). Regarding diagnostic imaging modalities, Doppler ultrasound and MRI were performed for all included patients. Thirty-seven patients underwent additional computed tomography (CT) scans for the delineation of the tumor extension. The majority of patients (71/95, 74.7%) were classified as Altman type I and type II tumors. The patients’ clinical characteristics are summarized in Table 1.

**Table 1:** The patients’ clinical characteristics

Parameters (total number of patients = 95)	Number of patients (%)
**Gender**	
Female	74 (78%)
Male	21 (22%)
**Age at diagnosis**	
Antenatal	24 (25%)
At birth or neonatal	52 (55%)
Late presentation	19 (20%)
**Associated anomalies**	
**No**	80 (84.2%)
**Yes**	15 (15.8%)
*Congenital heart diseases*	6
*Renal anomalies*	5
*Currarino syndrome*	4
**Altman classification**	
I	39 (41%)
II	32 (33.7%)
III	18 (19%)
IV	6 (6.3%)
**Type of perineal incision**	
Vertical elliptical with midline closure	49 (51.5%)
Classic chevron with transverse closure	27 (28.5%)
Chevron with a slight vertical extension	19 (20%)

### Management and after-care outcome

Surgical resection of the primary lesion and the coccyx was performed for all included cases. Eighty-eight patients (92.7%) were managed as elective scheduled procedures, whereas the remaining 7 were operated on as urgent interventions due to bleeding within the tumor. Out of the 7 patients, three were diagnosed antenatally, and they underwent urgent surgery just after delivery by cesarean section. The remaining four patients were diagnosed following birth, and during the preparation for elective surgery, hemorrhage within the tumor was discovered, and urgent surgical resection was performed. The urgent interventions for these patients were to avoid irreversible hypovolemic shock and circulatory failure. The surgical procedure was conducted via the perineal approach alone in 79% of all patients (*n* = 75). The remaining 20 children (21%) underwent surgery using a combined abdominoperineal approach to facilitate tumor excision. Resection of the abdominal component was done by laparotomy in 19 patients, whilst only one underwent complete laparoscopic excision of the abdominal component. Regarding the perineal incisions, vertical elliptical incision with midline closure was the commonest incision that was conducted in 49 patients (51.5%). Of them, 39 patients underwent a vertical closure in the natal cleft, whilst the remaining 10 cases had an inverted-Y-shaped closure to accommodate excess skin due to irregular tumors. The classic chevron incision with transverse closure was performed in 28.5% of patients (*n* = 27). A chevron incision with a slight vertical extension was adopted in 19 cases (20%). Concerning intraoperative complications, severe bleeding occurred during surgery in four patients. Surgical ligation, electrocautery, and sealing devices were successful in controlling the bleeding in three patients, whereas one patient underwent temporary packing and reexploration after 2 days. This patient had an iatrogenic injury to the external anal sphincter (additionally to severe bleeding) that was managed by surgical repair and proximal defunctioning colostomy to prevent perineal sepsis. Localized intraoperative tumor rupture happened in another three cases. There were no intraoperative deaths among the included patients. Three patients died in the first 10 days after surgery due to septic and metabolic complications. One of them underwent a second surgery to close a burst abdominal midline wound. Minor wound infection was encountered during the early follow-up period in 14 patients (14.8%). Eight patients (8.5%) had superficial postoperative wound dehiscence that was managed by local dressings and healed by secondary intention without further suturing. Two patients underwent a revision of the perineal scar during the follow-up period.

**Fig. 1:**
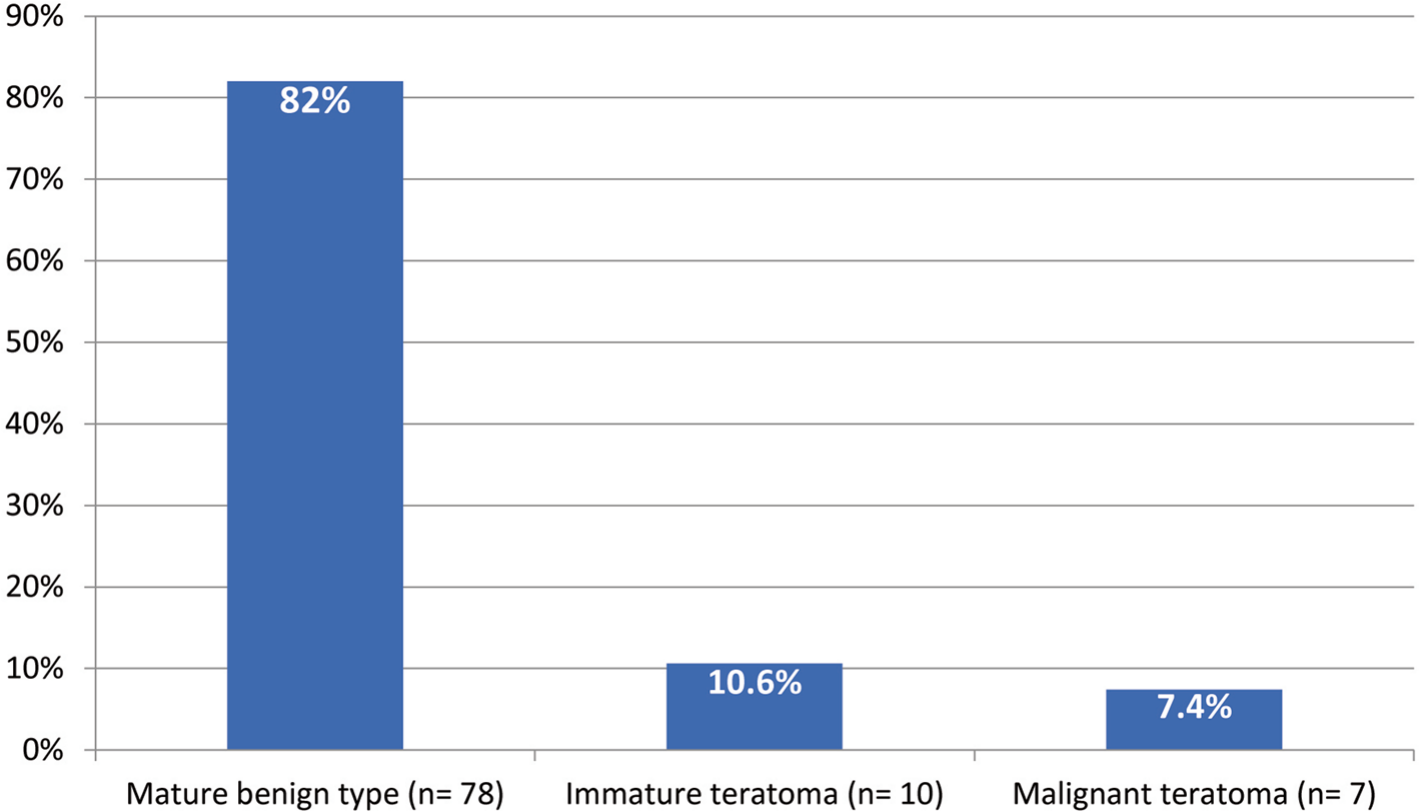
The distribution of pathological types among the included patients

**Fig. 2:**
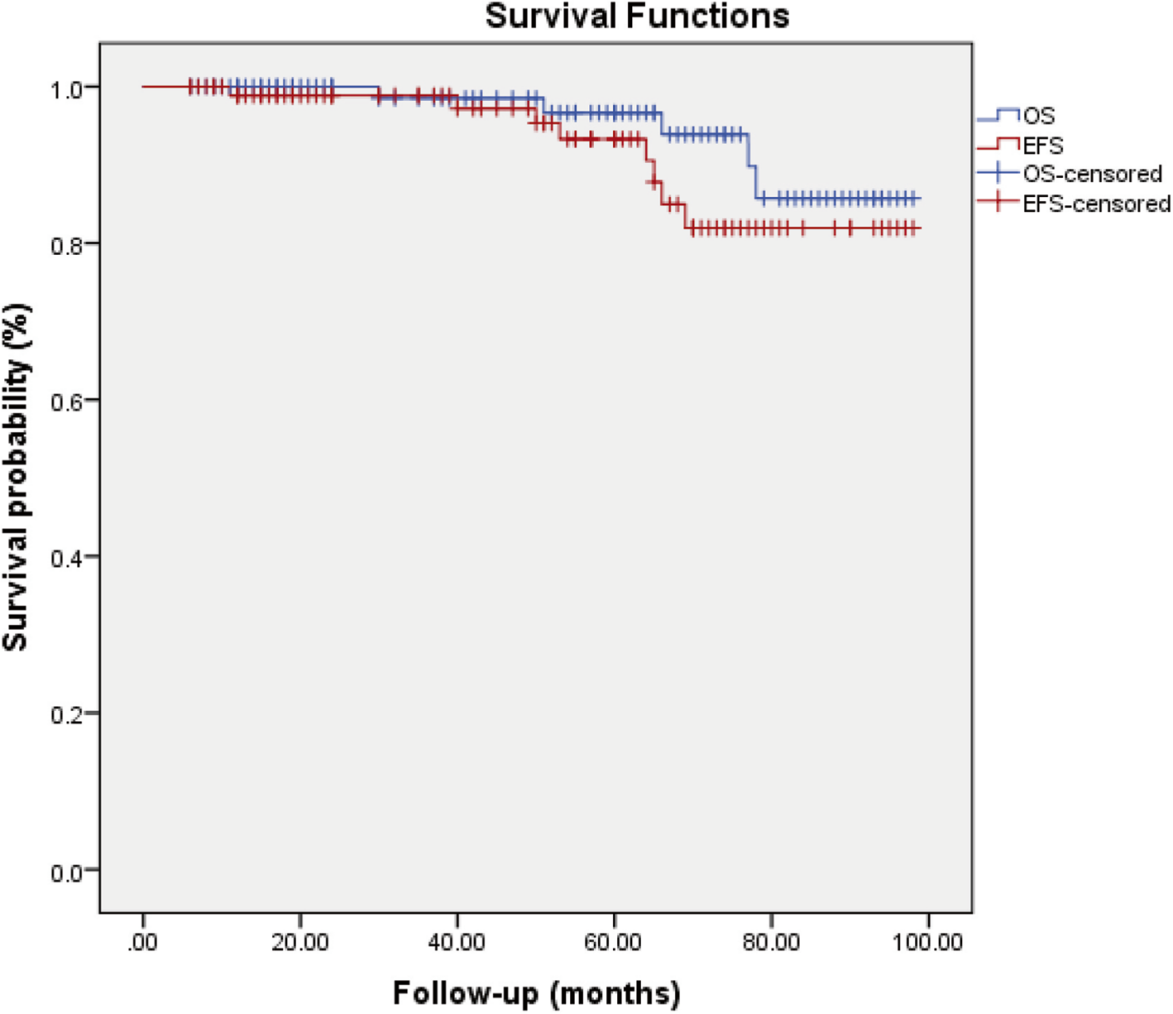
The overall and event-free survival of all patients

The majority of resected tumors were of benign mature type (*n* = 78, 82%), followed by immature teratoma and malignant teratoma. Figure 1 demonstrates the distribution of pathological types among the included patients. Platinum-based adjuvant chemotherapy was administered to the seven patients with confirmed malignant pathology. The follow-up duration ranged between 6 and 98 months (median: 57 months). Eight patients (8.5%) had a local malignant recurrence, and the diagnosis was based on imaging, raised level of serum AFP, and a biopsy. Four of them had previous malignant pathology, three patients were of the benign type, and one case had immature teratoma in the primary pathology. Chemotherapy was administered to all patients who had local recurrence, and two of them underwent a second surgical resection. Out of the eight patients; two patients died due to disease progression, whereas the remaining six had complete regression. At the end of follow-up time, five patients died, and the remaining 90 patients were alive and disease-free. The 5-year OS and EFS of all patients were 94% and 91%, respectively, as depicted in Fig. 2.

### Functional outcome

Seven patients (7.4%) had urological functional problems in the after-care follow-up. Five of them were Altman III, and the remaining two had Altman IV tumors. Out of the seven patients, two were diagnosed with an overactive bladder that was successfully managed using anticholinergic medications (oxybutynin 0.5 mg/kg twice daily). The urodynamic diagnosis of neurogenic bladder with normal capacity was established in five patients. Clean intermittent catheterization (CIC) was practiced in those patients. Two patients (who had Altman IV tumors) underwent further surgical procedures. Insertion of a suprapubic catheter was conducted in one of them, and the remaining patient underwent a Mitrofanoff catheterizable channel without bladder augmentation.

During clinical follow-up, constipation with or without fecal soiling was encountered in 12 patients (12.6%). Eight patients had Altman III tumors, whereas the remaining four were Altman II type. Out of the 12 patients; seven were managed using oral laxatives. Two patients had additional rectal irrigation besides laxatives. The remaining three patients underwent further surgery for the management of fecal incontinence in the form of an antegrade continence enema (ACE). Overall, four patients had both urinary and bowel functional problems, and all of them were Altman III/IV neoplasms.

## Discussion

The authors report the first Egyptian multicenter study concerning SCT to assess a national management strategy. This series highlighted the clinical characteristics and the surgical modalities for children with this uncommon neoplasm. Our findings with regard to female predominance, age groups at diagnosis, rates of associated congenital anomalies, and typical clinical presentations were in line with previous international publications [6, 19–21]. Antenatal diagnosis is crucial to avoid tumor rupture and to determine the timing and the approach of delivery. In our study, 25% of index patients were diagnosed antenatally. Two recent studies conducted in the UK and Japan declared that antenatal diagnosis was established in 44% and 41.4% of their patients, respectively [22, 23]. Such a difference mandates increasing the awareness of antenatal diagnosis of SCT among obstetricians in our country. Fetuses with SCT are at a high risk of developing perinatal morbidities, and the diagnosis before 20 weeks of gestational age is a predictor for potential mortality [24]. Thus, the delivery of any fetus with antenatal diagnosis of SCT should be delayed as possible to allow for further fetal maturation. However, intrauterine intervention for tumor resection is performed for diligently selected fetuses with progressive high-output cardiac failure [25].

Surgery is indicated for all children with SCT as originally described by Gross et al. [26]. Complete tumor resection, reconstruction of perineal muscles, and restoration of normal perineal and gluteal shape are the aims of the surgical procedure. In our study, the authors used three perineal incisions to allow excision of the lesion. The traditional chevron and modified chevron incisions were adopted in 28.5% and 20% of patients, respectively. Both incisions are still satisfactory due to achieving most of the procedure’s goals [27, 28]. However, the transverse incision is against the anatomical line of perineal muscles and the rectum leading to suboptimal access in addition to undesirable cosmetic scar with dog ear formation which is the principal disadvantage [28, 29]. Consequently, we performed the vertical elliptical incision with midline reconstruction in more than half of the patients due to its technical feasibility and aesthetic advantage over transverse incisions. The vertical incision was successfully adopted without any oncological or functional compromises in our series as well as another recent, international study [30]. Nevertheless, we believe that the choice of the perineal incision should be decided as per the preference of the operating surgeon.

The major intraoperative risk during tumor resection is iatrogenic injury to the vascular and visceral structures. We depended on bipolar cautery with great attention to secure median sacral vessels, and we used a Hegar dilator in the rectum to allow its identification. Dissection of the tumor was carefully performed from its lateral and inferior attachments to secure the anal sphincters. We strongly advocate that such operative strategies can significantly minimize vascular and/or visceral injuries through the procedure. This study demonstrated favorable surgical results, as operative complications were encountered in only seven patients (7.4%). Moreover, the mortality that occurred in two patients during the early postoperative period was due to nonsurgical etiology. Previous western studies documented excellent OS rates of SCT that ranged between 89 and 95% [19, 31]. Rescorla et al. also reported an OS of 90% in patients with malignant SCT who were managed using surgery and platinum-based chemotherapy [32]. Similarly, we achieved excellent OS either by surgery and observation approach for mature and immature types or multidisciplinary treatment strategy for malignant teratoma. Although the aforementioned favorable survival rates, tumor recurrence can be encountered in 2–35% of patients as reported previously [33, 34]. Incomplete resection, intraoperative tumor spillage, and malignant pathology are the potential risk factors for recurrence [1]. Local recurrence occurred in 8.5% of our index cases at a median follow-up of 57 months, and half of them had initially malignant tumors. Braungart et al. reported a local recurrence in 7% of their patients at a median follow-up of 60 months [22]. Padilla et al. documented that 13% of index cases developed recurrence with a median follow-up of 5 years [1]. Therefore, we advocate that all children with resected SCT should be closely monitored until adolescent age. Certainly, early detection of tumor recurrence and the administration of chemotherapy and/or a second surgery can be curative for those patients. Table 2 summarizes the outcomes of this study compared to the results from other previous studies.

**Table 2:** The outcomes of this study compared to the results from other previous studies

Study and year	Number of patients	Abdominoperineal approach (*n*)	Perineal incision: commonest (*n*)	Intraoperative complications (*n*)	Pathology (*n*)		Relapse (*n*)	Mortality (*n*)	Overall survival	Bowel dysfunction (*n*)	Bladder dysfunction (*n*)
					**Benign/immature**	**Malignant**					
**Current study (2023)**	95	20	Vertical; 49	7	88	7	8	5	94%	12	7
**Braungart (2023)** [22]	165	-	-	8	151	14	13	5	97%	52	22
**O'Shea (2022) **[30]	32	6	Vertical; 24	-	26	6	0	0	100%	1	4
**Masahata (2020)** [23]	29	7	Chevron; 29	3	28	1	0	1	96.6%	6	4
**Hambraeus (2019)** [35]	53	-	-	-	52	1	8	4	95.2%	19	24
**Padilla (2017)** [1]	40	-	Chevron; 40	-	38	2	5	2	95%	-	-
**Hambraeus (2016)** [31]	19	-	-	4	-	-	0	2	89%	-	-
**Hassan (2014)** [16]	20	3	Chevron; 17	2	19	1	2	1	95%	9	0
**Jan 2011 **[28]	19	0	Vertical; 19	0	14	5	0	0	100%	5	0
**Tailor (2009)** [36]	9	4	Chevron; 5	4	9	0	1	0	100%	1	3
**Gabra (2006)** [19]	33	8	Chevron; 33	3	27	6	3	2	94%	5	4

The assessment of long-term outcomes after resection of SCT is mandatory. Urinary incontinence and constipation with or without fecal soiling should be precisely evaluated, as early detection can avoid deterioration. These dysfunctions have been reported by previous single or multicentric international studies with rates of 20–50% [13, 19, 23, 35, 37, 38]. The difference of rates between these studies can be attributed to the variable number of patients and the duration of follow-up. The distortion of pelvic anatomy, stretching of muscles and nerves, and the manipulation during tumor resection may contribute to such adverse functional sequelae [22]. In our study, 20% of patients developed bowel or urological functional problems in the follow-up period. Medical therapy was effective in some children to control the symptoms; however, other patients required healthcare interventions such as CIC, suprapubic catheter insertion, Mitrofanoff procedure, and ACE devices. A team of pediatric surgeons subspecialized in oncology, urology, and colorectal services should be involved to improve the after-care quality of life for those patients. The authors of this study observed that patients with Altman III/IV tumor types were at a greater risk of encountering such long-term dysfunctions, and similar results were documented by others [19, 39]. We believe that these findings are crucial to be considered by the operating surgeon when discussing the procedure and obtaining consent from the patient’s guardian for surgery. The current study has a few limitations such as its retrospective design, consanguinity, and socioeconomic data of some patients were missed, different follow-up schedules between the participating centers, and some patients were followed up for a relatively short time. Eventually, this is the first Egyptian multicentric study regarding SCT, and the authors advocate that a national prospective registration system for these neoplasms is required in our country to improve future management and research.

## Conclusions

Favorable outcomes for SCT were achieved in our country during the last decade. Vertical elliptical incision with midline closure was the commonest perineal incision for tumor resection. During follow-up, 20% of patients developed urological or bowel dysfunctions that required medical and surgical treatment modalities to improve their quality of life.

## Data Availability

The datasets used and/or analyzed during the current study are available from the corresponding author on a reasonable request.
